# Optimized fluorescent labeling to identify memory B cells specific for *Neisseria meningitidis* serogroup B vaccine antigens *ex vivo*

**DOI:** 10.1002/iid3.3

**Published:** 2013-10-30

**Authors:** Nitya Nair, Ludovico Buti, Elisa Faenzi, Francesca Buricchi, Sandra Nuti, Chiara Sammicheli, Simona Tavarini, Maximilian WL Popp, Hidde Ploegh, Francesco Berti, Mariagrazia Pizza, Flora Castellino, Oretta Finco, Rino Rappuoli, Giuseppe Del Giudice, Grazia Galli, Monia Bardelli

**Affiliations:** 1 Novartis Vaccines & DiagnosticsSiena, Italy; 2 Whitehead Institute for Biomedical ResearchCambridge, Massachusetts; 3 University of Rochester, School of Medicine & DentistryRochester, New York; 4 Department of Biology, Massachusetts Institute of TechnologyCambridge, Massachusetts

**Keywords:** Antigen-specific memory B cells, flow cytometry, *Neisseria meningitidis* MenB, sortagging, vaccination

## Abstract

Antigen-specific memory B cells generate anamnestic responses and high affinity antibodies upon re-exposure to pathogens. Attempts to isolate rare antigen-specific memory B cells for in-depth functional analysis at the single-cell level have been hindered by the lack of tools with adequate sensitivity. We applied two independent methods of protein labeling to sensitive and specific *ex vivo* identification of antigen-specific memory B cells by flow cytometry: stringently controlled amine labeling, and sortagging, a novel method whereby a single nucleophilic fluorochrome molecule is added onto an LPETG motif carried by the target protein. We show that sortagged NadA, a major antigen in the meningococcal serogroup B vaccine, identifies NadA-specific memory B cells with high sensitivity and specificity, comparable to NadA amine-labeled under stringent reaction parameters in a mouse model of vaccination. We distinguish NadA-specific switched MBC induced by vaccination from the background signal contributed by splenic transitional and marginal zone B cells. In conclusion, we demonstrate that protein structural data coupled with sortag technology allows the development of engineered antigens that are as sensitive and specific as conventional chemically labeled antigens in detecting rare MBC, and minimize the possibility of disrupting conformational B cell epitopes.

## Introduction

Antigen-specific memory B cells generate anamnestic responses and high affinity antibodies upon re-exposure to bacterial and viral pathogens. The mechanisms through which memory B cells are involved in the generation and maintenance of long-term serologic memory remain unclear since protective antibody titers do not necessarily correlate with the number of memory B cells induced by infection and/or vaccination [1–4]. It is likely that both the quality and the size of the memory B cell pool are important determinants of the overall protective response to infection and/or vaccination.

Qualitative assessments of memory B cells *ex vivo* have been challenging due to their low frequency in peripheral blood [1,5]. As a consequence most studies have relied on expansion and conversion of memory B cells into antibody secreting cells by *in vitro* polyclonal stimulation with TLR ligands (CpG-2006, R848) and cytokines (IL-2, IL-10 or IL-6) for subsequent analysis by ELISpot or serial limiting dilution assay [1,2,6]. An alternative strategy has been to use fluorescently labeled proteins to identify antigen-specific MBC from mice and humans for qualitative analysis by flow cytometry [5,7]. However low signal to noise ratio is often observed due to low memory B cell frequencies and high background due to the fluorochrome itself [8,9].

Previous work has shown that dual antigen staining, in which tetanus (TT) or diphtheria (DT) toxoid were labeled with different fluorochromes, increased specificity and maintained sensitivity in the identification of TT- and DT-specific memory B cells as a double positive population by flow cytometry [5]. Dual antigen staining requires labeled antigens with equivalent affinities for the B cell receptor (BCR) to facilitate unbiased detection of memory B cell populations [8]. However most conventional labeling methods involve chemical attachment of fluorochrome molecules to accessible amine groups on the protein of interest [5,7], during which the positions and numbers of labeled amines, cannot be easily controlled. Furthermore, amine labeling may interfere with protein folding and disrupt conformational B cell epitopes at random, therefore skewing the selection of antigen-specific memory B cells for downstream analysis.

We describe two independent methods to fluorescently label protein antigens: conventional amine labeling with stringently controlled reaction parameters, and sortagging, a novel site-specific labeling method mediated by staphylococcal sortase A, in which a known number of nucleophilic fluorochrome molecules are added to LPTEG motifs expressed on the target proteins [10]. In both methods the degree of labeling is minimized.

As a model antigen we used *Neisseria* adhesin A (NadA), a major protein present in a multicomponent meningococcal serogroup B vaccine in advanced stage of development, and a virulence factor involved in meningococcus invasion and adhesion to epithelial cells [11,12]. NadA is an oligomeric coiled-coil adhesin with a trimeric structure and binding of NadA to human cells *in vitro* requires proper N-terminal domain folding and maintenance of its trimeric conformation [13,14]. Using sortagging we added a single fluorochrome molecule to the C-terminus of NadA so as to minimize potential conformation disruption, while for amine-labeling, we used the lowest protein to fluorochrome molar ratio that yielded high signal to noise intensity in FACS staining.

We demonstrate that amine-labeled and sortagged NadA allow *ex vivo* identification of all NadA-specific memory B cells by FACS in a mouse model of vaccination. Sortagged NadA performed as well as amine-labeled NadA prepared using controlled reaction parameters, in terms of sensitivity and specificity. Single antigen staining using either detection reagent was sufficient to thoroughly identify NadA-specific memory B cells among the total memory B cell population. In addition we distinguished the NadA-specific switched memory B cells induced by vaccination, from the background binding reactivity contributed by transitional and marginal zone splenic B cells with low affinity IgM receptors. We provide proof of concept that sortagged NadA can be used in dual staining with other antigens to identify memory B cells with distinct antigenic specificities. Fluorescent sortagged protein antigens therefore are improved alternatives to conventional chemically labeled baits due to the added benefits of quantitative site-specific labeling that permit better epitope preservation.

## Results

### Sortagging and amine-labeling of NadA with fluorochromes

**Figure 1:**
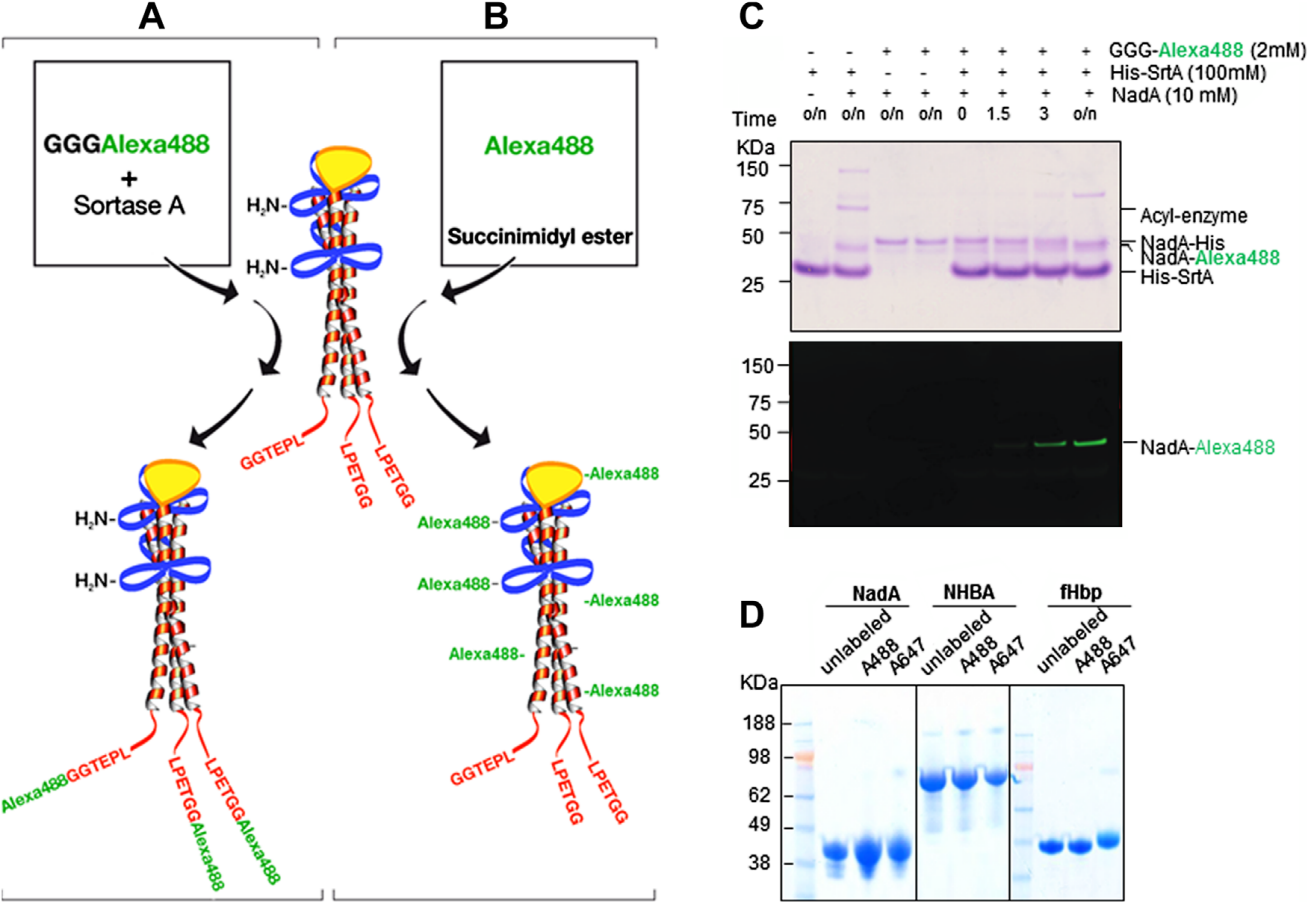
Sortagging and amine-labeling of NadA. (A) Model of the three-dimensional organization of NadA with dimeric and trimeric coiled-coil-rich stalk regions (blue, red) and globular head (yellow). (A) Sortagging (A) inserts fluorochrome molecules in a site-specific manner to the C terminus of the elongated coiled-coil-rich stalk region while (B) chemical labeling inserts flurochrome molecules at random to solvent accessible amine groups. (C) C-terminal NadA labeling using SrtA_Staph_. NadA with a C-terminal LPETGG followed by HA and His tag (Nad-His) (10 µM) was incubated with 100 µM SrtA with and without GGG-Alexa 488 (2 mM). The reaction was terminated at various times with Laemmli sample buffer, subjected to SDS–PAGE, and analyzed by Coomassie staining (upper panel) and fluorescent gel scanning (bottom panel). (D) Integrity of amine-labeled MenB vaccine antigens analyzed by SDS–PAGE and Coomassie staining.

According to the proposed three-dimensional model of NadA [14], sortagging inserted the Alexa fluorochrome to the C-terminus of the coiled-coil-rich stalk region of the protein (Fig. 1A). Conversely chemical labeling tagged the solvent accessible amine groups at random (Fig. 1B).

NadA equipped with the LPETG sortagging recognition motif followed by a C-terminal HA and His_(6)_ affinity handle were expressed and purified from *Escherichia coli*. Sortase A (srtA) containing an N-terminal His_(6)_ tag was produced as previously described [10]. The presence of the His tag on SrtA and NadA allowed to remove both SrtA and unreacted NadA after completion of the reaction. Overnight incubation of NadA with SrtA resulted in the formation of an acyl-enzyme intermediate (Fig. 1C, lane two). Addition of the gly-probe (respectively 3gly-488 or 3gly-647) drove the reaction to the transpeptidation product, and resulted in the site-specific labeling of NadA with A488 or A647 (Fig. 1C, lower insert and Fig. S1A). The overnight reactions (In) were loaded on a Ni-Nta column and SrtA and unreacted material were separated from labeled NadA, which was collected as a flow through (Fig. S1B, Ft lane). Proteins were dialyzed against PBS to remove excess probe (Fig. S1, lane D).

For amine-labeling, all the proteins were labeled at different protein to fluorochrome molar ratios. Conjugates labeled at the 10:1 molar ratio were selected as they had the lowest degree of labeling. Under these reaction conditions NadA, NHBA, and fHbp, were each tagged with roughly 1–2 mol of A488 or A647. HSA, chosen as a negative control for both fluorochromes in FACS staining, was tagged with roughly 3 mol of the same fluorochromes. Fluorescently labeled protein concentrations were >95% of input after removal of unlabeled fluorochrome by desalting column purification (Fig. 1D). All antigens were titrated to determine the concentration required to obtain adequate signal to noise intensity in FACS analysis (data not shown).

### Single antigen staining with amine-labeled or sortagged antigens identifies NadA-specific MBC

**Figure 2:**
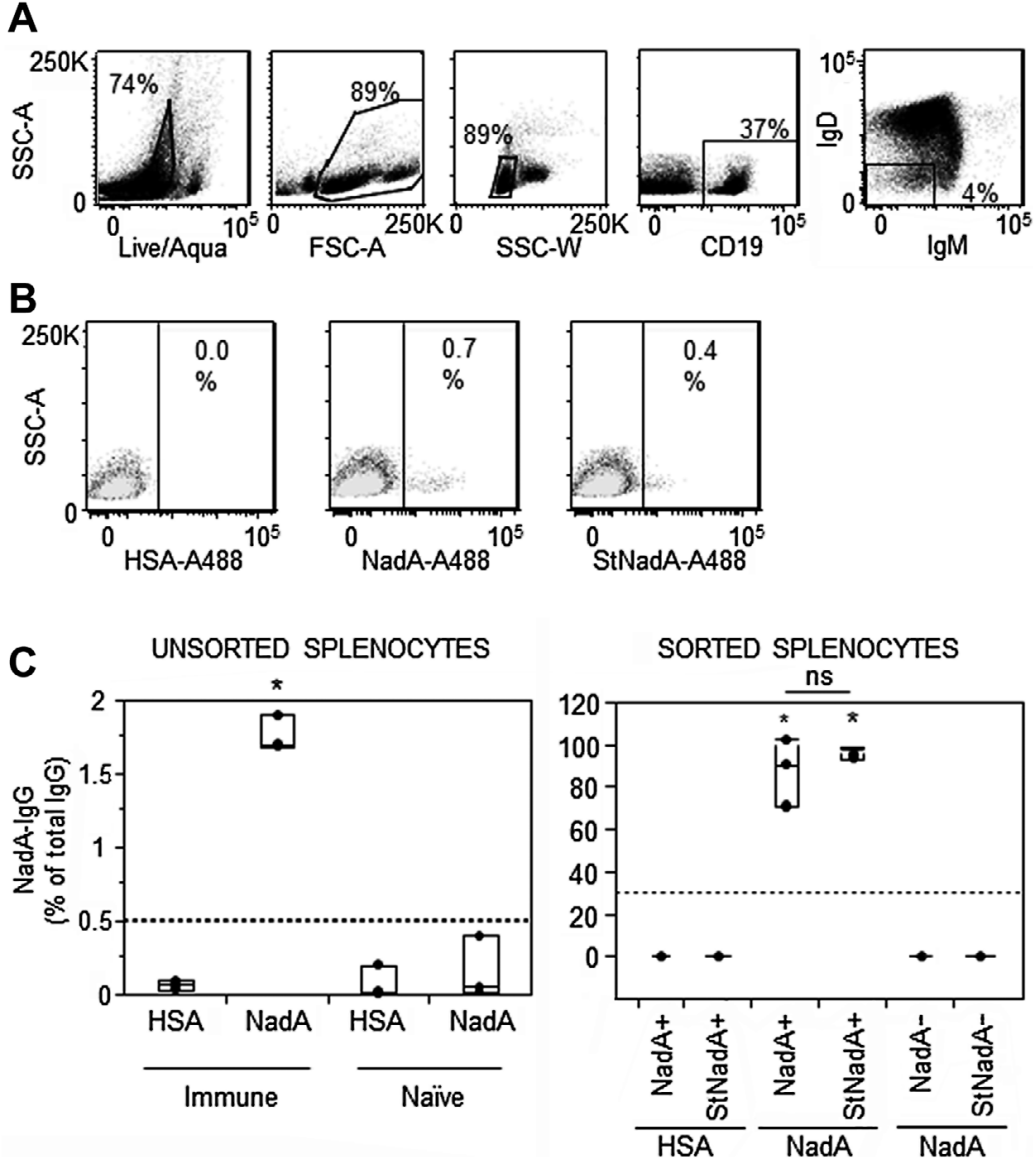
NadA-specific Ig-switched memory B cells are identifiable by FACS using amine-labeled and sortagged NadA-A488. (A) Murine splenocytes were gated based on Acqua live/dead staining, FSC × SSC morphology, single cells and CD19^+^IgD^−^IgM^−^ to identify Ig-switched memory B cells. (B) NadA-specific Ig-switched memory B cells were identified by single antigen staining using amine-labeled (NadA-A488) and sortagged (stNadA-A488) NadA, with gating based on staining in naïve mice (overlay in light gray) and HSA-A488 specificity controls. Data are representative of one of three different experiments. (C) NadA-A488^+^ and StNadA-A488^+^ were sorted together with NadA^−^ memory B cells and were cultured with CD19^−^ cells sorted from the same samples. NadA-specific IgG secreted by the NadA-specific memory B cells were determined by ELISpot, after five days of in vitro polyclonal stimulation with CpG-2006 and IL-2. Shown are mean percentages of NadA-specific IgG^+^ antibody secreting cells among total IgG^+^ antibody secreting cells in cultures of unsorted immune and naïve splenocytes (left), or in cultures of NadA+, NadA−, StNadA+ or StNadA−IgD−IgM− cells sorted from immune mice (right). Results are from three independent experiments performed with splenocytes pooled from four mice per group in each experiment. Box plots represent median values and ranges. The asterisks indicate samples with significantly greater frequencies of NadA-specific IgG as compared to all the others (*P*-value <0.05 by the Tukey–Kramer test; ns: not statistically significant).

To verify whether it was possible to identify B cells binding to NadA through BCR-specific interactions, splenocytes from naïve mice or mice immunized with serotype B meningococcal antigens were pooled and stained with either amine- (NadA-A488) or sortagged- (StNadA-A488) NadA. Distinct B cells bound to NadA and expressing class switched Ig receptors (IgD^−^IgM^−^) were identified in immune mice using either of the labeled baits. Stringent gating of NadA-specific switched memory B cells was set based on the background signal observed in negative control splenocytes from immune mice stained with the HSA-A488 (Fig. 2A and B). The same results were obtained with A647-labeled baits, however, these conjugates exhibited high background in staining even following dialysis and purification and were therefore not used.

To verify whether NadA-specific memory B cells, identified using NadA-A488 and StNadA-A488, were able to produce NadA-specific antibodies, NadA^+^ memory B cells and NadA^−^ memory B cells were sorted from immune mice, co-cultured with CD19^−^ cells, and stimulated *in vitro* for five days with CpG-2006 and IL-2 to generate antibody secreting cells. A fraction of unsorted splenocytes was also cultured as described to determine the degree of enrichment of NadA-specific IgG^+^ secreting cells in the NadA^+^ sorted population.

ELISpot results from three different experiments (Fig. 2C), showed that NadA-specific IgG^+^ antibody secreting cells generated in cultures of unsorted splenocytes accounted for roughly 1.8% (range: 1.7–1.9%) of all IgG^+^ antibody secreting cells detected in immune mice. Conversely, NadA-specific IgG^+^ antibody secreting cells were not detected in cultures of splenocytes from naïve mice (Fig. 2C).

Highly enriched proportions of NadA-specific IgG^+^ antibody secreting cells were found in cultures of NadA^+^ B cells, identified and sorted with either the NadA-A488 bait (range: 71–100%) or with the StNadA-A488 (range: 94–96%). No significant differences were found between frequencies of NadA-specific IgG^+^ antibody secreting cells generated by the two NadA-binding B cell populations identified with either bait, which in both cases, were more than 50-fold greater than in cultures of unsorted immune splenocytes (Fig. 2B). Remarkably, NadA-specific IgG^+^ antibody secreting cells were never observed in cultures of B cells sorted as NadA^−^ (NadA− and StNadA− in Fig. 2B) and cells secreting antibodies reactive with HSA were detected at extremely low and comparable frequencies in all samples (Fig. 2B).

These results indicate that a single antigen staining strategy, using either NadA-A488 or StNadA-A488, thoroughly identifies NadA-specific Ig-switched memory B cells. Amine-labeled NadA-A488 consistently stained NadA-specific memory B cells at slightly higher frequency (mean 0.8%, range: 1.1–0.5%) compared to sortagged NadA-A488 (mean 0.5%, range: 0.7–0.3%), perhaps due to the addition of a greater number of fluorochrome molecules during the labeling reaction. Nevertheless the difference in the frequency of NadA-specific IgG^+^ antibody secreting cells identified by ELISpot after staining and sorting with either reagent was never statistically significant and no NadA-specific IgG^+^ antibody secreting cells were identified in the NadA− sorted B cells.

### Single and double staining approaches identify NadA-specific B cells with comparable specificities

**Figure 3:**
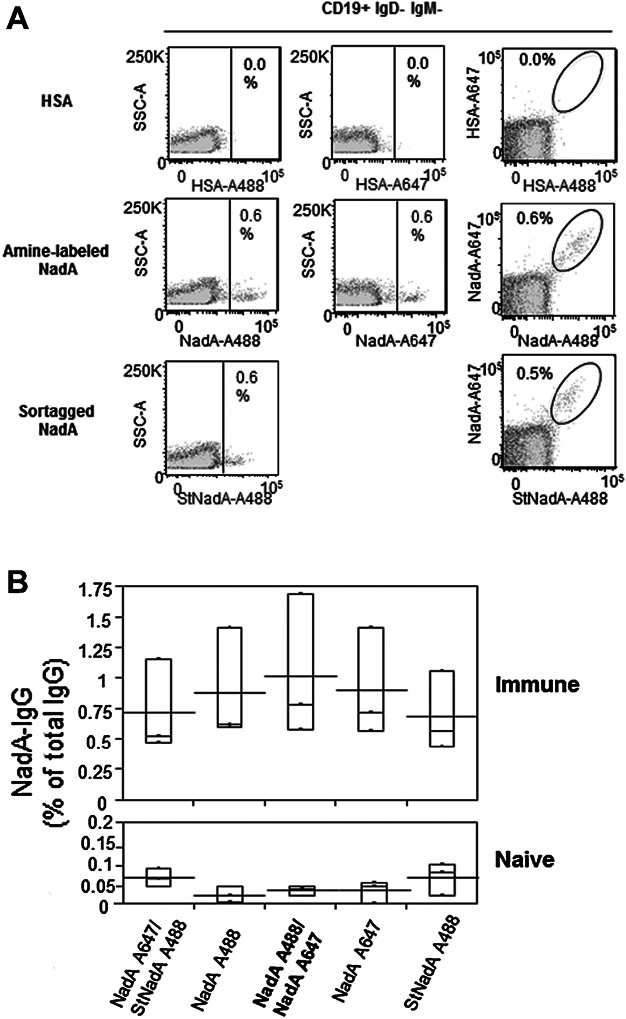
Single antigen staining is sufficient to identify NadA-specific switched memory B cells. (A) Representative staining of NadA-specific memory B cells identified by single or dual antigen staining strategies with amine-labeled or sortagged NadA-Alexa fluorochrome conjugates. Gating of NadA-specific memory B cells was based on staining in naïve mice (overlay in light gray) and HSA specificity controls. (B) For each staining strategy, the percentage of NadA-specific memory B cells among total switched memory B cells is shown in immune (top) and naïve (bottom) mice after subtracting background HSA staining. Results are from three independent experiments performed with splenocytes pooled from four mice per group. Box plots depict median values and ranges; the box-crossing lines depict the means.

We compared the frequencies of NadA-specific MBC identified by a single fluorescent bait (amine-labeled or sortagged) to those identified by combining two baits that were amine-labeled with different fluorochromes. The results from three independent experiments showed that the frequencies of switched B cells binding to one or two baits were comparable (Fig. 3A). The mean frequency of NadA-specific memory B cells identified by dual antigen staining did not differ from those observed in samples stained with a single amine-labeled or sortagged NadA bait (*P*-values for comparison across all groups and between each pair of samples was always >0.9 by the Tukey–Kramer test) (Fig. 3B). In addition, to verify whether the signal to noise ratios were higher in samples stained with two NadA baits than in those stained with a single NadA we compared the ratios of mean fluorescence intensities measured for each bait in the negative and positive gates using the one-tailed Wilcoxon test. The results of this analysis showed that differences in signal to noise ratios in each fluorescence channel were not statistically significant (data not shown).

Overall these results show that the use of a single amine-labeled or sortagged antigen is sufficient to identify NadA-specific Ig class-switched memory B cells.

### Staining with a single antigen bait is sufficient to monitor changes in NadA-specific B cells induced by vaccination

**Figure 4:**
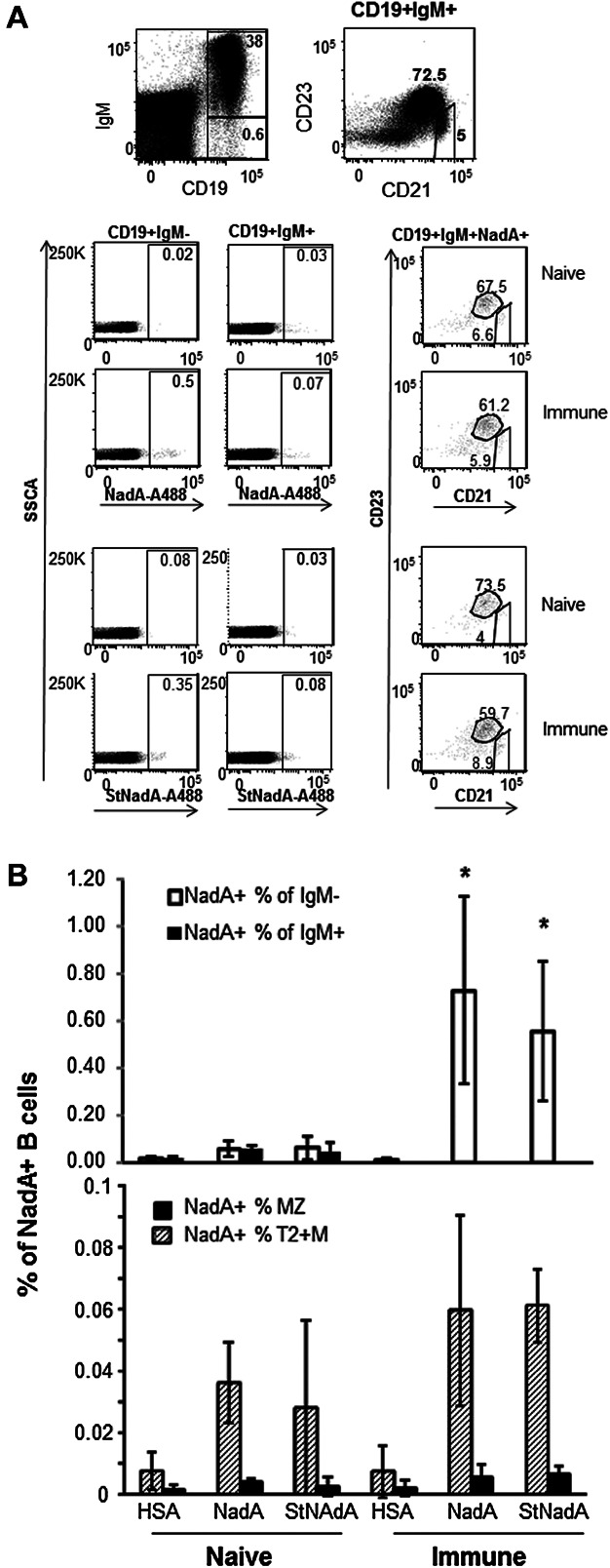
Most NadA-binding B cells are Ig-class switched memory B cells specifically induced by vaccination. NadA-binding reactivity distributed among type-2 transitional, mature-naïve and marginal zone B cells does not change upon vaccination. (A) Upper panels: Gating strategy to identify splenic transitional type 2 plus mature-naïve (T2 + M) (CD19^+^IgM^+^CD21^+^CD23^+^) and MZ (CD19^+^IgM^+^CD21^hi^CD23^−^) B cells. Lower panels: representative dot plots showing that most of NadA-specific B cells were Ig-switched CD19^+^IgM^−^ B cells present in immune but not naïve mice. NadA+ IgM^+^ B cells were present at comparable frequencies in naïve and immune mice and mostly distributed across T2 + M and MZ subsets. (B) Mean frequencies of NadA-binding B cells among CD19^+^IgM^−^ and CD19^+^IgM^+^ populations (top), and among MZ and T2 + M B cell subsets (bottom) in naïve and immune mice. Shown are the mean values (±standard deviations) from three independent experiments, each performed with four mice per group. The asterisks indicate statistically significant differences between mean frequencies of HSA^+^ IgM^−^ B cells and either NadA^+^ or StNadA^+^ IgM^−^ B cells in immune mice (*P* < 0.01 by the Tukey–Kramer test).

We analyzed the distribution of NadA-binding B cells across switched MBC memory B cells (CD19^+^IgM^−^), transitional type 2 and mature-naïve (T2 + M) (CD19^+^IgM^+^CD21^+^CD23^hi^) and MZ (CD19^+^IgM^+^CD21^hi^CD23^low^) B cells in immune and naïve mice (Fig. 4A). The results from three independent experiments showed that vaccination induced a significant increase in the frequency of Ig class switched NadA-binding B cells (mean values of NadA^+^ CD19^+^IgM^−^ in immune and naïve mice were 0.65% and 0.06% of total B cells, respectively; *P* < 0.05 by the Student's *t*-test) (Fig. 4b). In contrast, comparable frequencies of NadA^+^ cells were detected in the T2 + M and MZ B cell subsets of naïve and immune mice. Most of the NadA reactivity in CD19^+^IgM^+^ B cells was detected in T2 + M B cells, and to a lesser extent, in MZ B cells (Fig. 4B). The binding pattern of amine-labeled and sortagged NadA were always comparable independently of the B cell subset analyzed (Fig. 4B) (*P*-values for the comparisons between NadA-amine labeled and NadA-Sortagged by Tukey Kramer test were 0.89; 1 and 0.99 in Ig-switched memory B cells, T2 + M and MZ B cells, respectively).

These results show that single antigen staining with either amine-labeled or sortagged NadA have comparable binding patterns across T2 + M and MZ B cells, suggesting that such background binding is mainly due to the intrinsic cross-reactive capacity of T2 and MZ B cells, and not to changes in NadA antigenic determinants introduced by the conjugation reactions.

### MBC specific for two vaccine antigens can be detected in a single staining reaction

**Figure 5:**
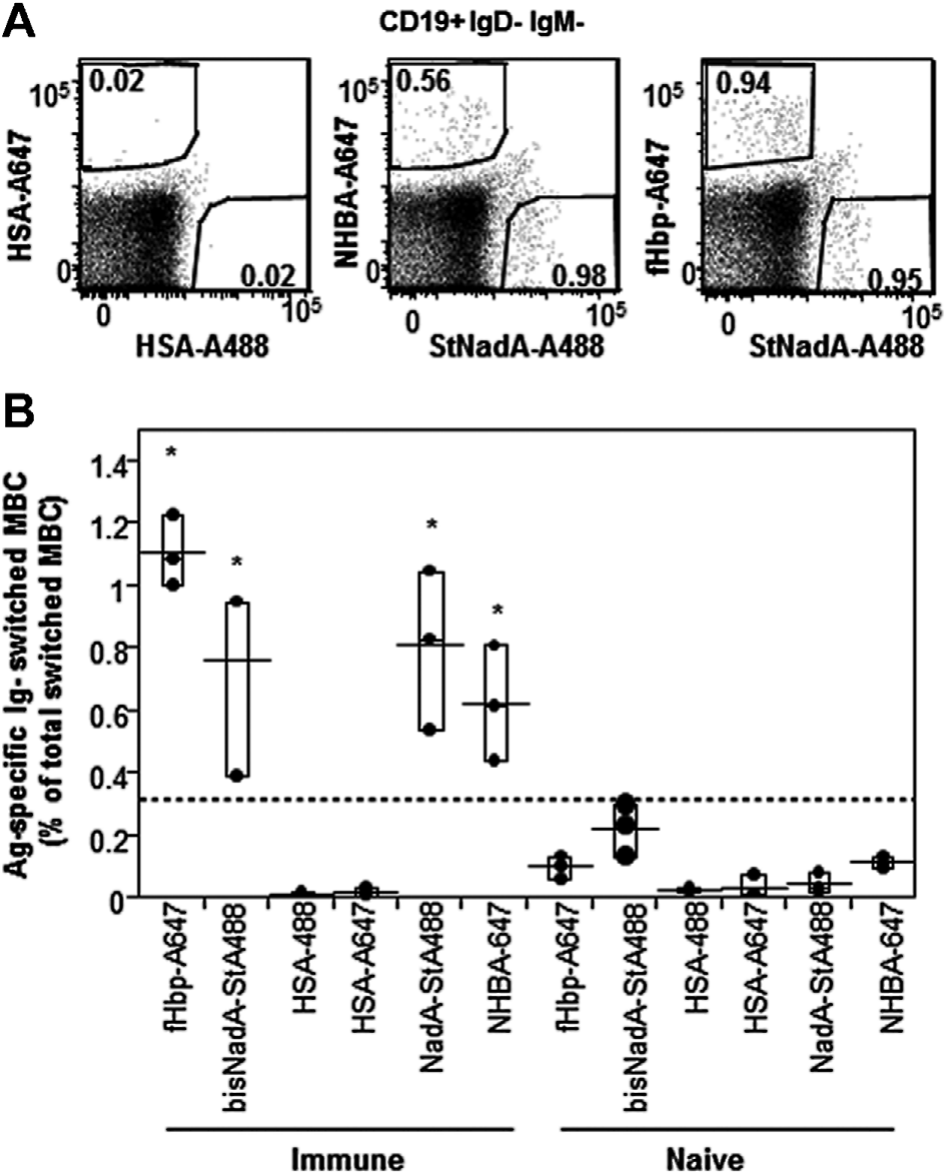
Simultaneous detection of class switched memory B cells specific to two meningococcal serogroup B vaccine antigens from a single FACS staining reaction. (A) Representative staining of switched memory B cells specific for different MenB vaccine antigens in combination with StNadA-Alexa488. Negative control samples were stained with labeled HSA. (B) Results of the same experiment repeated in three different immune mice and in control naïve mice show that both fHbp and NHBA-specific B cells can be detected in combinations with StNadA in a double staining approach. The asterisks indicate statistically significant differences in mean frequencies of ^+^ B cells between immune and naïve mice (*P*-value ≤0.01 by the Tukey–Kramer test).

Finally, we investigated the possibility of combining StNadA-A488 in dual staining with other antigens used to immunize mice, namely, NHBA and fHbp conjugated to A647. Distinct populations of Ig-switched memory B cells binding to NHBA, fHbp or to NadA were consistently observed in immune mice (Fig. 5A) and at significantly higher frequencies than in naïve mice (all *P* ≤ 0.01 by the Tukey–Kramer test) (Fig. 5B). Minimal background staining was observed in naïve mice and in HSA specificity controls. The frequency of NadA-specific switched memory B cells detected using StNadA-A488 in combination with either fHbp or NHBA was consistent (Fig. 5B), supporting the reliability of this approach. These results demonstrate that sensitive and specific detection of two diverse antigen-specific memory B cell populations is feasible in a single staining reaction.

## Discussion

The identification of antigen-specific B cells by flow cytometry is technically challenging due to low memory B cell frequencies and to high background [8,9]. In this study we showed the application of two labeling methods for sensitive and specific identification of vaccine-induced NadA-specific switched IgD^−^IgM^−^CD19^+^ murine B cells by flow cytometry. We used conventional amine-labeling, in which reaction parameters were stringently controlled to reduce the degree of labeling and to increase the signal to noise ratio in FACS. In addition we use sortagging, a novel labeling method in which a quantified number of fluorochrome molecules were tagged to NadA in a site-specific manner. For both methods, the degree of labeling was minimized and the integrity of the labeled antigens was preserved.

We demonstrated that single antigen staining with amine-labeled and sortagged NadA is sufficient to thoroughly identify all NadA-specific IgD^−^IgM^−^CD19^+^ B cells with high sensitivity and specificity in a mouse model of meningococcal serogroup B vaccination. Memory B cells that bound amine-labeled and sortagged NadA by FACS, when sorted and polyclonally activated, yielded comparable frequencies of NadA-specific IgG^+^ antibody secreting cells by ELISpot to those sorted using the amine-labeled bait, while the remaining flow through fraction of NadA-non-binding B cells contained no detectable NadA-specific IgG^+^ antibody secreting cells. Although there are no specific surface markers to define murine memory B cells, the identified population can be considered *bona fide* memory B cells, due to the expression of a class switched BCR. In addition ELISpot analysis of the NadA^+^ populations identified with either bait could be stimulated to differentiate into an almost pure population of antibody secreting cells secreting IgG specific for NadA (around 90%). Of note, the frequencies of NadA-specific memory B cells detected by FACS analysis among CD19^+^IgD^−^IgM^−^ population (0.7% using NadA-A488) were comparable to the frequencies of NadA-specific IgG produced by antibody secreting cells as determined by ELISpot assay, which is a traditional method to assess memory B cell responses to various antigens. It should also be noted that the amine-labeling reaction parameters were optimized in this study so as to minimize the degree of labeling, while preserving antigen integrity and maintaining high signal to noise ratio in FACS staining. These parameters for conventionally labeled antigens used in FACS analysis of memory B cells are seldom reported in the literature. It is feasible that, under conditions of excessive or unoptimized labeling, alterations to B cell conformational epitopes may increase, emphasizing the advantage of using the sortagging approach.

As Moody and Haynes [8,15] point out, the use of dual antigen-specific labeling in selecting for the cells labeled by both fluorochromes is useful to reduce the background attributed to the fluorochrome itself, however, this strategy will not eliminate background entirely. In addition, if the two detection reagents possess differential affinities for the BCR, some cells may selectively bind one of the two reagents with greater affinity than the other, hence underestimating the full extent of the specific antigen-BCR interaction. In addition, the quantity of labeled antigen used for detection of antigen-specific B cells is important since high concentrations can result in aggregates that cross-link multiple B cells through their BCR, thus reducing the number of single cells for subsequent sorting and phenotypic analysis. This phenomenon is particularly important when tetramers or multimers are employed to identify antigen-specific B cells and is avoided by the use of single antigen baits. It should be noted that the mean frequency of NadA-specific memory B cells identified in our studies by dual antigen staining with amine-labeled NadA (65 events/million MBC, range 39–116) was slightly higher than in samples stained with a single bait (55 events/million MBC, range 30–93) or by dual antigen staining with sortagged NadA (46 events/million MBC, range 22–82), however these differences were not statistically significant. As mentioned, NadA-specific IgG^+^ antibody secreting cells were never observed in cultures of sorted memory B cells that stained negative for NadA, supporting our observation that a single Ag bait is sufficient to identify all antigen-specific B cells.

The validated single antigen staining permits the use of the spared fluorescence channel to simultaneously analyze memory B cells with distinct specificities to other meningococcal antigens such as NHBA and fHbp, in combination with NadA, in a single staining reaction. This feature may be particularly useful in clinical trial settings where sample volumes are limited, such as in the analysis of the infant immune response to meningococcal vaccination.

While the majority of class switched NadA-binding B cells in the murine spleen were specifically induced by vaccination, NadA-labeled baits bound to a remarkable number of IgM^+^ B cells, among which a high proportion of transitional and naïve B cells (T2 + M) and MZ B cells were detected. T2 + M and MZ B cells are known to play an important role in the initial, rapid, low-affinity antibody response to bacteria and viruses, through the production of poly-reactive antibodies [16–18].

In conclusion we demonstrate the use of amine-labeled and sortagged NadA for highly sensitive and specific identification of murine antigen-specific memory B cells by single antigen flow cytometric staining. Sortagged NadA in this study yielded equivalent sensitivity and specificity in identifying and isolating NadA-specific murine memory B cells compared to conventional amine-labeled NadA despite the addition of just a single fluorochrome molecule to the target protein in the sortagged conjugate, as opposed to multiple fluorochrome molecules in the amine-labeled conjugate. The benefit of the sortagging approach compared to conventional amine-labeling, could be the enhanced preservation of conformational epitopes and the ability to introduce quantified fluorochrome molecules into specific antigenic regions while maintaining native conformation of the target antigen. The method is broadly applicable to a range of recombinant proteins and particularly relevant to complex, multimeric antigens. For example, fluorescent sortagging of influenza hemagglutinin and neuraminidase proteins facilitated the direct visualization of virus release from host cell surface as well as the release of newly formed virus particles [19]. We present sortagging as an alternative to amine-labeling, which could be particularly advantageous when coupled with antigen structural and epitope mapping data. We demonstrate the simultaneous detection of memory B cells with distinct specificities to other meningococcal antigens from a single antigen staining reaction, particularly relevant for the analysis of human antigen-specific memory B cells in clinical trial specimens. Having established proof of concept in methodology using a murine model of vaccination, sortagging of vaccine antigens can be applied to the flow cytometric analysis of human antigen-specific memory B cells in vaccine trials. In this context the enhanced preservation of B cell epitopes may facilitate repertoire analysis in large cohorts of subjects, immunogen discovery and rational vaccine design.

## Materials and Methods

### Fluorescent labeling of NadA

NadA was labeled with Alexa 488 (A488) and Alexa 647 (A647) fluorochromes using sortagging and amine-labeling strategies (Fig. 1A).

#### Synthesis of GGGK-A647 and GGGK-A488 probes and sortagging of NadA

The GGGK peptide was synthesized by Fmoc-based solid phase peptide synthesis on Rink Amide Resin (Novabiochem, Darmstadt, Germany). The Fmoc-protected peptide was cleaved from the resin and the protective group was removed by treatment with 2.5 mL of 95:3:2 TFA-TIPS/H_2_O (5×, 15 min each). The combined cleavage solutions were concentrated, dissolved in methanol, and precipitated with cold diethyl ether. The Fmoc-GGGK peptide was mixed with 0.5 equiv. of A647 or A488, and 4 equiv. of DIPEA (Sigma, St. Louis, MO, USA) in anhydrous DMSO and incubated for 6 h at room temperature. The Alexa 488 peptide was purified by reversed-phase HPLC on a Waters Delta Pak C18 column (MeCN:H_2_O gradient mobile phase containing 0.1% TFA). The A647 peptide was similarly purified on a Waters 5PE column. Peptide identity was confirmed for the A488 peptide by MALDI-TOF MS (matrix: sinapinic acid), [M+] = 831.16, obs = 832.862; both HPLC peaks contained this mass. The molecular weight and activity of A647 nucleophile was inferred by setting up a test transpeptidation reaction on a LPETG tagged GFP substrate as described [10], and observing the mass change in the transpeptidation product by ESI-MS on a Micromass LCT mass spectrometer with a Waters Symmetry 5 μm C8 column (MeCN:H_2_O (0.1% formic acid) gradient mobile phase). The predicted molecular weight for the A647 peptide is 1155.06, obs = 1155.0.

For sortagging 10 μM of NadA with a C-terminal LPETGG followed by HA and His tag (Novartis Vaccines & Diagnostics, Siena, Italy), were incubated with 100 μM sortase A (SrtA) (from *Staphylococcus aureus*) in the presence of GGG-A488 or GGG-A647 (2 mM). Sortagged NadA conjugates were purified from residual unlabeled material and SrtA using Ni-NTA resin followed by dialysis of the flow through material.

#### Amine-labeling of NadA with A488 and A647

NadA, NHBA, fHbp, and HSA were labeled with mixed carboxylic acid and succinimidyl ester isomers of A488 and A647 according to manufacturer's protocol (Molecular Probes, Carlsbad, CA, USA). Briefly, 1 mg of protein was incubated at a molar ratio of 5:1 and 10:1, of fluorochrome to protein, for 2 h at room temperature in the presence of 100 mM NaHCO_3_. The reaction was terminated with 50 mM Tris–HCl for 10 min at room temperature. Unlabeled fluorochrome was removed using a desalting spin column (Thermo Scientific, Waltham, MA, USA). The degree of labeling (DOL) was calculated as follows: *A*_max_ × MW/[protein] × *ε*_dye,_ where *A*_max_ is the absorbance of the protein-fluorochrome conjugate (at 495 nm for Alexa 488 and 650 nm for Alexa 647), MW is the molecular weight of the protein (Da), *ε*_dye_ is the extinction coefficient of the dye at its maximum absorbance (*ε*_A488_: 7.10 × 10^4^, *ε*_A647_: 2.39 × 10^5^) and the protein concentration is expressed in mg/mL. Protein concentration of fluorescently labeled antigens was determined by bicinchoninic acid assay (BCA) (Thermo Scientific).

### SDS–PAGE analysis of fluorescently labeled NadA conjugates

The integrity of fluorescently labeled proteins was assessed by SDS–PAGE. Briefly, 5 μg of each protein was analyzed in reducing and denaturing conditions by 4–12% gradient acrylamide gel electrophoresis (Criterium XT, Bio-Rad, Hercules, CA, USA) before and after labeling. Protein bands were visualized by EZ Blue™ Gel Staining Reagent (Sigma, St. Louis, MO, USA) containing Comassie blue.

### Mice immunization

Six weeks old BALB/c mice were injected subcutaneously three times (two weeks apart) with 200 μL of PBS containing 20 μg each of the serotype B meningococcal antigens NHBA, fHbp, NadA, and 10 μg of meningococcal outer membrane vesicles (OMV) formulated in Al(OH)_3_. All proteins used for mouse immunizations were not fluorescently labeled. Control mice received only Al(OH)_3_ at the same time points. Three weeks after the final immunization mice were euthanized and their spleens were harvested. The study was approved by the local Animal Welfare Body and was conducted according to the animal welfare guidelines of Novartis Vaccines & Diagnostics (AEC permission number 2009/09 approved by the Italian Ministry of Health on 15 June 2009).

### Flow cytometric identification of NadA-specific MBC

NadA-specific class-switched memory B cells were identified by flow cytometry using a modified version of a previous protocol [5]. Splenocytes from four mice per group were pooled stained with the Acqua live/dead cell stain (Invitrogen, Carlsbad, CA, USA) for 20 min at room temperature and then blocked with 20% normal rabbit serum (NRS) for 20 min at 4 °C. Roughly 10 × 10^6^ splenocytes were stained per reaction for 1 h at 4 °C with the following monoclonal antibodies (mAbs): anti-CD19 PE (clone 1DR, BD Biosciences, San Jose, CA, USA), anti-IgD eFluor v450 (clone 11–26, eBiosciences, San Diego, CA, USA), anti-IgM PerCP (clone R6-60.2, BD Biosciences), anti-CD21/CD35 APC (clone 7G6, BD Biosciences), anti-CD23 PB (clone B3B4, Biolegend, San Diego, CA, USA). To identify antigen-specific memory B cells, samples were stained with 0.3 μg of sortagged or amine-labeled NadA-A488 or NadA-A647. The optimal amount of fluorescently labeled protein to be used was determined in titration experiments on naïve and immune mice. Negative control samples were stained with HSA amine-labeled with A488 or A647. Cells were analyzed on BD FACS Canto II or sorted using BD FACS Aria II and at least 3 × 10^6^ events were acquired per sample. The analysis was performed using the FlowJo software v9.1 (Tree Star, Inc., Ashland, OR, USA) with gating based on live cells, FSC × SSC characteristics, singlets and CD19^+^ expression. Immunoglobulin-class-switched MBC were identified as CD19^+^IgD^−^IgM^−^.

### ELISpot analysis of NadA-specific memory B cells identified by flow cytometry

ELISpot analysis of NadA-specific memory B cells identified by flow cytometry was performed using a modified version of a previous protocol [20]. Pooled splenocytes from immune mice were stained in parallel with sortagged or amine-labeled NadA-A488. NadA-binding CD19^+^IgD^−^IgM^−^ as well as CD19^−^ splenocytes were sorted from immune mice, mixed at the ratio of 0.5:100 and cultured in vitro at 3 × 10^5^ cells in 200 μL of complete medium with CpG (2.5 μg/mL) (Primm) and IL-2 (500 U/mL) (Novartis). After five days, cells were harvested and plated into ELISpot plates (MultiScreen HTS-HA, Millipore, Billerica, MA, USA) previously coated with 100 μL of PBS containing 10 μg/mL of NadA, or HSA, or 5 μg/mL of a goat anti-mouse IgG antibody (Jackson Immuno Research, Westgrove, PA, USA). Plates were blocked for 2 h at room temperature with PBS containing 10% fetal bovine serum, and then incubated with biotin-conjugated goat anti-mouse IgG (Southern biotech, Birmingham, AL, USA), followed by horseradish peroxidase-conjugated streptavidin (Endogen, Rockford, IL, USA). Plates were washed and developed with the AEC substrate kit (Sigma). Spots of antibody secreting cells were counted using the UV Spot ELISpot plate Analyzer (CTL, OH, USA) and the Immunospot software v5.09 (CTL).

### Statistics

Statistical analyses were performed using JMP software (version 8.0.1). Differences between the means of log10-transformed NadA-memory B cell frequencies were analyzed by the Tukey–Kramer test for multiple comparisons. Differences between mean ratios of Ag-specific to background fluorescence intensities (MFI) were analyzed by the one-tail Wilcoxon test, as implemented in the *stats* package of R version 2.14 (http://www.r-project.org/). *P* values ≤0.05 were considered significant.

## SUPPORTING INFORMATION

**Figure S1.** NadA labeling with SrtA_Staph_ purification. (A) On a preparative scale 10 µm NadA-His was labeled with SrtA using either the GGG-Alexa 488 or GGG-Alexa 647 probes (2 mM). The different reactions were analyzed by SDS–PAGE to confirm labeling of NadA. (B) NadA-Alexa 488 and NadA-Alexa 647 were separated from residual unlabeled material and srtA (In) using Ni-NTA resin and the collected flow-through (FT) was dialyzed. The fractions were analyzed as described in (A).
