# Supplementary figures and images for: Optimized fluorescent labeling to identify memory B cells specific for Neisseria meningitidis serogroup B vaccine antigens ex vivo
Source: Immun Inflamm Dis. 2013 Oct 30;1(1):3–13. doi: 10.1002/iid3.3 (PMC4217542; doi:10.1002/iid3.3)

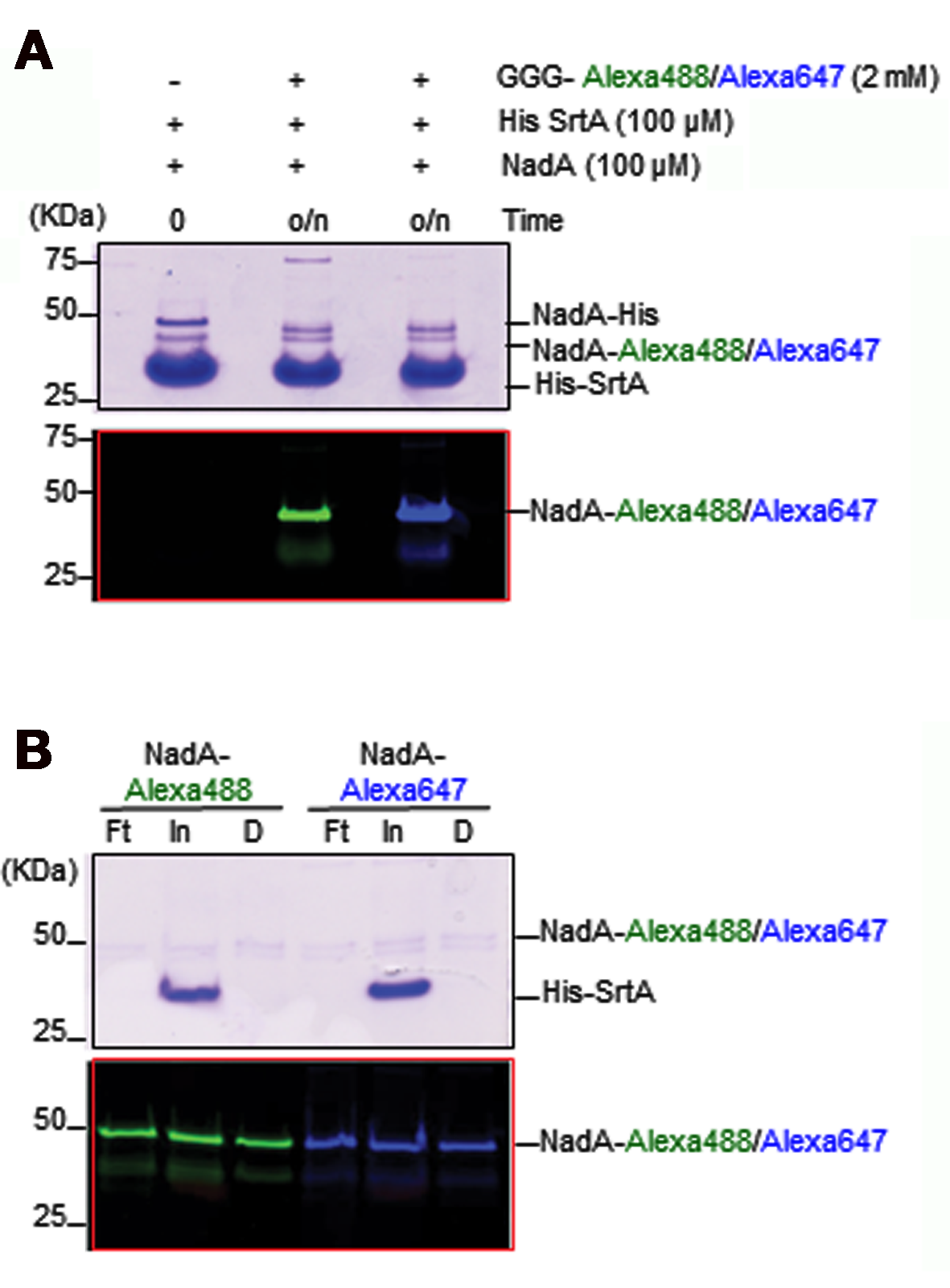

Supplement: Supplementary file 1 — Figure S1. NadA labeling with SrtAStaph purification. (A) On a preparative scale 10 µm NadA-His was labeled with SrtA using either the GGG-Alexa 488 or GGG-Alexa 647 probes (2 mM). The different reactions were analyzed by SDS–PAGE to confirm labeling of NadA. (B) NadA-Alexa 488 and NadA-Alexa 647 were separated from residual unlabeled material and srtA (In) using Ni-NTA resin and the collected flow-through (FT) was dialyzed. The fractions were analyzed as described in (A). [file iid30001-0003-SD1.tif]
